# Association between non-high-density lipoprotein cholesterol to high-density lipoprotein cholesterol ratio and sarcopenia in individuals with cancer: a cross-sectional study

**DOI:** 10.1186/s12944-024-02205-x

**Published:** 2024-07-16

**Authors:** Ran He, Youjun Ye, Qilei Zhu, Changsheng Xie

**Affiliations:** 1 The First School of Clinical Medicine, Zhejiang Chinese Medical University, Hangzhou, China; 2 The Third Clinical Medical College, Zhejiang Chinese Medical University, Hangzhou, China; 3 Department of Medical Oncology, The First Affiliated Hospital of Zhejiang Chinese Medical University (Zhejiang Provincial Hospital of Traditional Chinese Medicine), Hangzhou, China

**Keywords:** NHHR, Cancer, Sarcopenia, Lipid metabolism, HDL-C, NHANES

## Abstract

**Background:**

Cancer and sarcopenia are both closely related to lipid metabolism, but the relationship between lipid metabolism and patients with cancer and sarcopenia has not been thoroughly studied. The non-high-density lipoprotein cholesterol to high-density lipoprotein cholesterol ratio (NHHR) is a reliable measure of lipid metabolism. The purpose of this study was to determine the possible relationship between the NHHR and sarcopenia in individuals with cancer.

**Methods:**

Data from the National Health and Nutrition Examination Survey (NHANES) database for individuals with cancer, with and without sarcopenia was analyzed using weighted multiple regression equations, weighted regression cubic spline (RCS) analysis, and weighted subgroup analysis.

**Results:**

In total, 1,602 individuals with cancer were included, of whom 17.1% had sarcopenia. In Adjusted Model 2, the occurrence of sarcopenia was found to be significantly associated with a higher NHHR in cancer (95% confidence interval [CI]:1.01–1.39, *P* = 0.036). Individuals with high a NHHR had a 2.09-fold higher risk of developing sarcopenia in comparison to those with a low NHHR (95% CI:1.12–3.92, *P* = 0.022). RCS analysis further identified a U-shaped non-linear relationship between females with cancer and the muscle index. Subgroup analysis indicated that sex was a significant stratifying factor, whereas age, race, marital status, smoking and drinking habits, and history of cardiovascular disease, arthritis, hypertension, and diabetes had no significant impact.

**Conclusion:**

From the perspective of lipid metabolism, the NHHR may serve as an indicator for monitoring and preventing the occurrence of sarcopenia in individuals with cancer, particularly for females with cancer who appear to have greater sensitivity.

## Background

Sarcopenia is an, age-related loss of muscle mass characterized by an accelerated loss of muscle function [1]. According to surveys, sarcopenia affects 5–10% of the general population, worldwide, [2] and is showing an increasing trend. The prevalence of sarcopenia is high in individuals with cancers, ranging from 15 to 50% [3]. Many characteristics of cancer overlap with those of aging, which is the most important factor i*n* the prevalence and progression of sarcopenia. Anti-tumor therapy can accelerate the mutation frequency of skeletal muscles at the genetic level, accelerating genetic aging and leading to sarcopenia [4]. Chronic inflammation and oxidative stress secondary to cancer are the high-risk factors for sarcopenia [5]. At the molecular level, skeletal muscle cells secrete myokines, such as interleukin 6, oncostatin M, and irisin. These myokines regulate the tumor microenvironment through crosstalk with other tissues, affecting cancer cell proliferation, apoptosis, and metabolic reprogramming [6]. Sarcopenia in individuals with cancer can lead to abnormal myokine metabolism, causing lipid and glucose metabolism disorders and chronic inflammation, ultimately promoting cancer progression [7]. From a clinical perspective, individuals with cancer and sarcopenia often face an increased risk of falls and disabilities, ultimately compromising their quality of life and significantly shortening their survival [8]. Therefore, as a common comorbidity in individuals with cancer and a significant factor that increases the risk of disease progression, it is necessary to urgently explore the potential factors leading to sarcopenia in these individuals for clinical monitoring and prevention.

The non-high-density lipoprotein cholesterol (non-HDL-C) to high-density lipoprotein cholesterol (HDL-C) ratio (NHHR) is a novel composite index that can be used to evaluate the lipid spectrum that causes atherosclerosis. Previous studies have demonstrated that the NHHR offers superior diagnostic performance in predicting the risk of cardiovascular diseases, some liver and kidney diseases, and metabolic syndrome, compared to traditional cholesterol indices [9–12]. Additionally, a recent study has reported a correlation with cancer and mortality [13], which holds significance in assessing cancer prognosis [14].

Several studies have reported on sarcopenia and abnormalities of lipid metabolism [15]. Multiple cross-sectional investigations have revealed an inverse relationship between the triglyceride/HDL-C ratio and relative grip strength, indirectly showing that lipid metabolism monitoring-related indicators reflect muscle loss [16–18]. When investigating lipoprotein subfraction levels in individuals with sarcopenia, a significantly elevated level of non-HDL-C was found, indicating a strong relationship between sarcopenia and abnormalities in lipid metabolism [19]. Moreover, it has been reported that individuals with cancer and sarcopenia have varying degrees of abnormal lipid metabolism, a higher body mass index (BMI), and a lower level of HDL-C [20]. It has been suggested that lipid metabolism-related parameters, such as BMI, low-density lipoprotein cholesterol (LDL-C), and total cholesterol (TC), within the normal reference range may assist in preventing sarcopenia [21].

However, while some studies have confirmed the vital function of lipid metabolism in the prevalence and development of sarcopenia in patients with cancer, others have also highlighted the significant role of high-density lipoproteins in sarcopenia [22, 23]. Nevertheless, the association between the NHHR in individuals with cancer and an elevated risk of sarcopenia has not been fully elucidated. Therefore, utilizing National Health and Nutrition Examination Survey (NHANES) data, this study aimed to determine the association between the NHHR and sarcopenia in individuals with cancer. The study also aims to provide reference data for monitoring and preventing sarcopenia in individuals with cancer from the perspective of lipid metabolism.

## Method

### Study cohort

All individuals included in this study were participants in NHANES. This database extensively surveys the nutritional and health status of adults and children in the United States (US) through laboratory tests, physical examinations, and questionnaires. Through weighting, reflecting its stratified multistage probability sampling, NHANES participants are more typical of the US population [24].

**Fig. 1:**
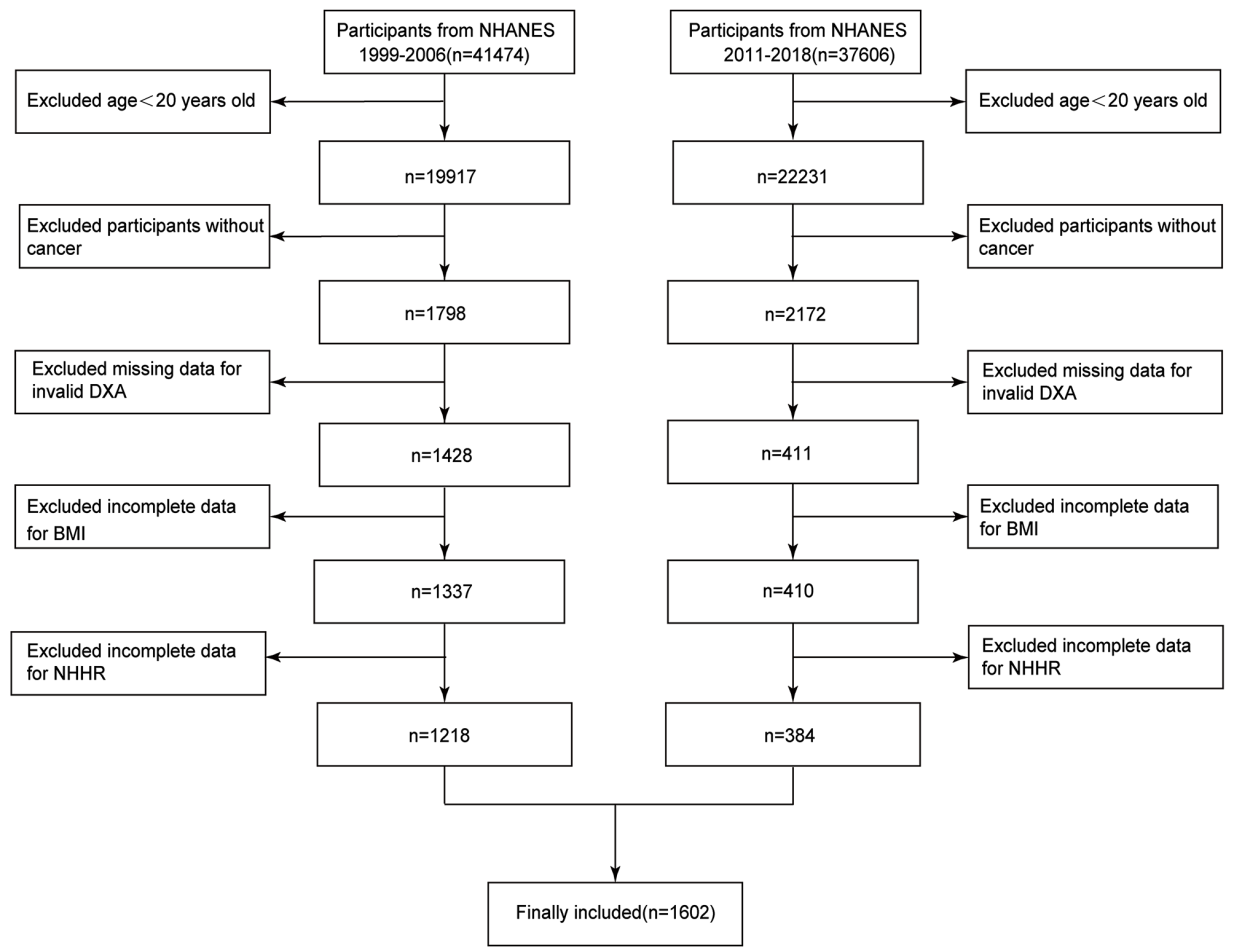
Study flowchart

Individuals included in this study were selected from participants included in the NHANES database from 1999 to 2006 and 2011 to 2018, with 41,474 and 37,606 individuals, respectively. Exclusion criteria were: <20 years; individuals without cancer; incomplete dual-energy X-ray absorptiometry (DXA); no skeletal muscle mass and BMI measurements; no TC and HDC-C values. After application of the exclusion criteria a total of 1,602 individuals were ultimately included (Fig. 1).

### Exposure definition

The NHHR served as the exposure variable in this study. Additionally, the NHHR was categorized into three levels, low, medium, and high, based on tertiles, for subsequent statistical analysis. The specific method involved ordering the NHHR values of all the participants in the sample in ascending order. The 33.3% and 66.6% percentiles were then calculated, dividing the data into three groups: low (below 2.26), medium (between 2.26 and 3.42), and high (above 3.42). This approach facilitated a more detailed analysis and is applicable to any data distribution.

### Outcome definition

The presence or absence of sarcopenia among the participants was the primary outcome measure of this study, defined by the sarcopenia index. Individuals in the NHANES databases from 1999 to 2006 and 2011 to 2018 underwent DXA measurements. The lean body mass of the four limbs was summed to determine the appendicular skeletal muscle mass. The muscle index was calculated by dividing the mass of the appendicular skeletal muscle (kg) by the BMI (kg/m^2^). Sarcopenia was considered to be present if the muscle index was < 0.789 for males and < 0.512 for females [25].

### Covariates

All possible confounding factors that might affect the relationship between the NHHR in individuals with cancer and the development of sarcopenia were thoroughly examined. Age and duration of moderate-intensity physical activity were included as continuous variables, whereas sex, race, income-to-poverty ratio, alcohol habit, marital status, smoking habit, educational attainment, history of heart failure, coronary disease, stroke, arthritis, hypertension, and diabetes were included as categorical variables. BMI, TC, and HDL-C, which are used to define the exposure variables and outcome measures, were not included as covariates to avoid potential bias due to collinearity. The time engaged in moderate-intensity physical exercise included both work and leisure time, standardized to minutes per week. The poverty-to-income ratio (PIR) divided the cohort into poverty (< 1) and non-poverty (≥ 1) groups. An alcohol habit is defined by the question “Have you ever had five or more drinks every day?” while a smoking habit is defined by the question, “Do you now smoke cigarettes?“.

### Statistical analysis

All statistical analyses considered sample weights and were conducted using “survey” in R for weighted calculations. For covariates with missing values, “mice” in R was used to impute missing values through multiple imputation methods. For a small number of participants (less than 1%) who refused to answer or had unknown responses to the questionnaire, a simple imputation strategy was used. Specifically, this study employed mode imputation based on the frequency distribution of valid responses to handle these missing data points.

Counts and (percentages) were used for categorical variables and the mean and (Standard Deviation) was used for continuous variables. To compare baseline characteristics across various groups in the overall cohort, weighted chi-square tests and weighted one-way ANOVA tests were used for categorical and continuous variable analyses, respectively.

Weighted multivariate logistic regression was conducted to assess the association between the NHHR in individuals with cancer and sarcopenia, incorporating three different models (unadjusted model, Adjusted Model 1, and Adjusted Model 2) to determine whether an independent association exists. The NHHR was categorized into low, medium, and high groups to assess whether there was a significant trend in the outcome measures. Weighted regression cubic spline (RCS) analysis was performed on the fully adjusted model to obtain dose-response curves between the NHHR, and sarcopenia and muscle index, examining the non-linear relationship between the NHHR, and sarcopenia and muscle index.

To further explore the potential factors influencing the association between the NHHR and sarcopenia, weighted regression analysis was conducted on the fully adjusted model, with age and physical activity transformed into categorical variables. Additionally, they were included as effect modifiers along with PIR, educational attainment, marital status, alcohol habit, smoking habit, coronary disease, arthritis, hypertension, and diabetes as potential influencing covariates in the subgroup analysis to determine if there were significant interactions among the different categories.

The unadjusted model was not adjusted for the covariates. Adjusted Model 1 was adjusted for sex, age, race, marital status, poverty status, and educational attainment. Building on Adjusted Model 1, Adjusted Model 2 was further adjusted for moderate-intensity physical activity, alcohol habit, smoking habit, heart failure, coronary disease, stroke, arthritis, hypertension, and diabetes.

Statistical significance was set at *P* < 0.05 and R 4.2.3 was used to perform all statistical analyses.

## Results

### Baseline characteristics

**Fig. 2:**
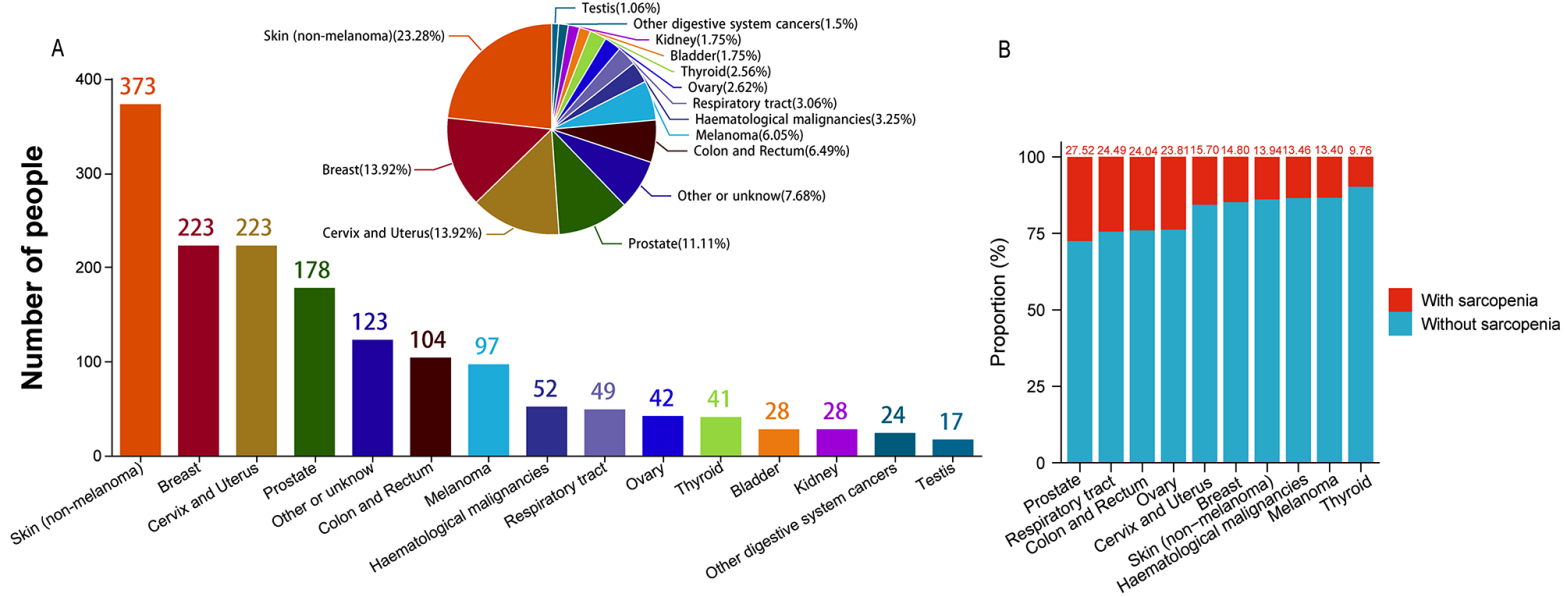
(**A**) Distribution of cancer cohorts among study participants. (**B**) Prevalence of sarcopenia among cancer cohorts

There were 1,602 individuals from the NHANES database between 1999 and 2006 and 2011–2018 who satisfied the inclusion requirements (Table 1). Among them, 42.88% were male and 57.12% were female, with a mean age of 60.78 (15.68) years. The prevalence of sarcopenia in individuals with cancer was 17.1%. There were significant differences between groups with and without sarcopenia for age, race, educational attainment, BMI, alcohol habit, smoking habit, history of heart failure, stroke, arthritis, hypertension, diabetes, coronary disease, HDL-C, and the NHHR. Individuals with sarcopenia and cancer were older, predominantly white non-Hispanics, had a higher education level of college or above, higher BMI, an alcohol habit, less smoking, and a higher prevalence of heart failure, stroke, arthritis, hypertension, diabetes, and coronary disease. They also had lower HDL-C and NHHR levels. Further statistical analyses of specific cancer types among the participants revealed 373 cases of non-melanoma skin cancer, 223 cases of breast cancer, 223 cases of cervical and uterine cancer, and 178 cases of prostate cancer. These four cancer types represented the largest percentages in this study. Non-melanoma skin cancer had the highest percentage (23.28%), whereas testicular cancer had the lowest percentage (1.06%). Other digestive system cancers, including liver cancer, stomach cancer, and esophageal cancer, were grouped under “Other digestive system cancers” due to their smaller sample sizes. Cancers with sample sizes smaller than that of testicular cancer, as well as those reported as “Other” or “Unknown,” were categorized under “Other or unknown,” (Fig. 2(A)). Additionally, the percentages of individuals with sarcopenia among the predominant cancer types were determined; with prostate, respiratory tract, colorectal, and ovarian cancers having relatively higher percentages (Fig. 2(B)).

**Table 1:** Baseline study population characteristics (weighted)

Characteristic	Total (n = 1602)	Without Sarcopenia (n = 1328)	With Sarcopenia (n = 274)	*P*-value
Gender (%)				0.1145
Male	687.00 (42.88%)	542.00 (40.81%)	145.00 (52.92%)	
Female	915.00 (57.12%)	786.00 (59.19%)	129.00 (47.08%)	
Age (year)	60.777 ± 15.657	59.078 ± 15.512	69.007 ± 13.638	< 0.0001
Age (%)				< 0.0001
< 60	759.00 (47.38%)	684.00 (51.51%)	75.00 (27.37%)	
≥ 60	843.00 (52.62%)	644.00 (48.49%)	199.00 (72.63%)	
Race(%)				0.0004
Mexican American	132.00 (8.24%)	87.00 (6.55%)	45.00 (16.42%)	
Other Hispanic	58.00 (3.62%)	44.00 (3.31%)	14.00 (5.11%)	
Non-Hispanic White	1156.00 (72.16%)	959.00 (72.21%)	197.00 (71.90%)	
Non-Hispanic Black	193.00 (12.05%)	183.00 (13.78%)	10.00 (3.65%)	
Other Race	63.00 (3.93%)	55.00 (4.14%)	8.00 (2.92%)	
Educational attainment (%)				0.0007
Less Than 9th Grade	162.00 (10.11%)	108.00 (8.13%)	54.00 (19.71%)	
9-11th Grade	199.00 (12.42%)	159.00 (11.97%)	40.00 (14.60%)	
High School Grad/GED or Equivalent	374.00 (23.35%)	304.00 (22.89%)	70.00 (25.55%)	
College or above	867.00 (54.12%)	757.00 (57.00%)	110.00 (40.15%)	
Marital status (%)				0.4248
Married or Living with Partner	592.00 (36.95%)	495.00 (37.27%)	97.00 (35.40%)	
Divorced or Living without Partner	1010.00 (63.05%)	833.00 (62.73%)	177.00 (64.60%)	
PIR (%)				0.6761
< 1	225.00 (14.04%)	190.00 (14.31%)	35.00 (12.77%)	
≥ 1	1377.00 (85.96%)	1138.00 (85.69%)	239.00 (87.23%)	
Body mass index (kg/m2)	28.523 ± 6.384	28.053 ± 6.164	30.801 ± 6.929	< 0.0001
Body mass index (%)				< 0.0001
< 25	507.00 (31.65%)	459.00 (34.56%)	48.00 (17.52%)	
≥ 25, ≤ 30	548.00 (34.21%)	453.00 (34.11%)	95.00 (34.67%)	
> 30	547.00 (34.14%)	416.00 (31.33%)	131.00 (47.81%)	
Moderate activity (min/week)	434.508 ± 668.490	444.360 ± 683.469	386.753 ± 589.338	0.8022
Moderate activity (%)				0.6882
< 150	667.00 (41.64%)	542.00 (40.81%)	125.00 (45.62%)	
≥ 150, ≤ 300	314.00 (19.60%)	261.00 (19.65%)	53.00 (19.34%)	
> 300	621.00 (38.76%)	525.00 (39.53%)	96.00 (35.04%)	
Alcohol habit (%)				0.0099
Yes	282.00 (17.60%)	211.00 (15.89%)	71.00 (25.91%)	
No	1320.00 (82.40%)	1117.00 (84.11%)	203.00 (74.09%)	
Smoking habit (%)				0.0003
Yes	513.00 (32.02%)	462.00 (34.79%)	51.00 (18.61%)	
NO	1089.00 (67.98%)	866.00 (65.21%)	223.00 (81.39%)	
Heart Failure (%)				0.0047
Yes	101.00 (6.30%)	73.00 (5.50%)	28.00 (10.22%)	
No	1501.00 (93.70%)	1255.00 (94.50%)	246.00 (89.78%)	
Coronary diease (%)				< 0.0001
Yes	128.00 (7.99%)	95.00 (7.15%)	33.00 (12.04%)	
No	1474.00 (92.01%)	1233.00 (92.85%)	241.00 (87.96%)	
Stroke (%)				0.0003
Yes	107.00 (6.68%)	72.00 (5.42%)	35.00 (12.77%)	
No	1495.00 (93.32%)	1256.00 (94.58%)	239.00 (87.23%)	
Arthritis (%)				0.0251
Yes	694.00 (43.32%)	553.00 (41.64%)	141.00 (51.46%)	
No	908.00 (56.68%)	775.00 (58.36%)	133.00 (48.54%)	
Hypertension (%)				0.0010
Yes	765.00 (47.75%)	606.00 (45.63%)	159.00 (58.03%)	
No	837.00 (52.25%)	722.00 (54.37%)	115.00 (41.97%)	
Diabetes (%)				0.0011
Yes	269.00 (16.79%)	207.00 (15.59%)	62.00 (22.63%)	
No	1333.00 (83.21%)	1121.00 (84.41%)	212.00 (77.37%)	
HDL-C (mg/dL)	54.492 ± 17.362	54.922 ± 17.610	52.405 ± 15.975	0.0050
TC (mg/dL)	203.130 ± 44.285	203.389 ± 44.155	201.876 ± 44.971	0.5004
NHHR	3.041 ± 1.420	3.017 ± 1.411	3.156 ± 1.461	0.0189
NHHR Tertiles (%)				0.0201
Low level	534.00 (33.33%)	459.00 (34.56%)	75.00 (27.37%)	
Medium level	534.00 (33.33%)	433.00 (32.61%)	101.00 (36.86%)	
High level	534.00 (33.33%)	436.00 (32.83%)	98.00 (35.77%)	

**Table 2:** The association between NHHR and sarcopenia in individuals with cancer (weighted)

	Unadjusted Model	Adjusted Model 1	Adjusted Model 2
	OR (95%CI), *P*-value		
NHHR	1.16 (1.02, 1.33), 0.027	1.21 (1.05, 1.14), 0.012	1.18 (1.01, 1.39), 0.036
NHHR Tertiles			
Low level	Reference	Reference	Reference
Medium level	1.75 (1.04, 2.96), 0.037	1.72 (1.03, 2.88), 0.038	1.55 (0.92, 2.62), 0.101
High level	2.19 (1.16, 4.11), 0.016	2.23 (1.19, 4.21), 0.013	2.09 (1.12, 3.92), 0.022
*P* for trend	0.014	0.013	0.022
Cancer subgroups			
Skin Cancer (non-melanoma)	1.04 (0.86, 1.25), 0.695	1.12 (0.89, 1.41), 0.326	0.99 (0.79, 1.23), 0.896
Breast Cancer	1.57 (1.24, 1.98), < 0.001	1.68 (1.26, 2.23), < 0.001	1.70 (1.25, 2.30), 0.001
Uterine and Cervical Cancer	1.12 (0.82, 1.54), 0.457	1.26 (0.82, 1.94), 0.285	0.86 (0.54, 1.37), 0.501
Prostate Cancer	0.80 (0.58, 1.11), 0.179	0.66 (0.35, 1.25), 0.214	0.66 (0.35, 1.25), 0.182

**Table 3:** Association between NHHR and sarcopenia in subgroups (weighted)

NHHR	Sarcopenia		
Subgroup	OR(95%CI)	*P*-value	*P* for interaction
Gender			0.006
Male	1.02(0.85, 1.23)	0.84	
Female	1.41(1.17, 1.70)	< 0.001	
Age			0.138
< 60	1.24(1.01, 1.53)	0.04	
≥ 60	0.95(0.78, 1.15)	0.57	
PIR			0.457
< 1	1.43(0.98, 2.08)	0.06	
≥ 1	1.16(1.00, 1.35)	0.05	
Educational attainment			0.238
Less Than 9th Grade	1.07(0.69, 1.68)	0.72	
9-11th Grade	0.93(0.64, 1.37)	0.71	
High School Grad/GED or Equivalent	1.03(0.76, 1.39)	0.86	
College or above	1.25(1.01, 1.54)	0.04	
Moderate activity			0.087
< 150	1.11(0.93, 1.31)	0.24	
≥ 150, ≤ 300	1.01(0.65, 1.58)	0.96	
> 300	1.56(1.22, 1.99)	< 0.001	
Marital status			0.771
Married or Living with Partner	1.18(0.95, 1.48)	0.13	
Divorced or Living without Partner	1.18(0.98, 1.43)	0.08	
Alcohol habit			0.177
Yes	1.08(0.85, 1.36)	0.53	
No	1.31(1.08, 1.59)	0.01	
Smoking habit			0.564
Yes	1.16(0.98, 1.38)	0.08	
No	1.30(0.96, 1.76)	0.09	
Coronary diease			0.971
Yes	1.08(0.59, 1.98)	0.71	
No	1.20(1.02, 1.41)	0.03	
Arthritis			0.12
Yes	1.30(1.06, 1.60)	0.01	
No	1.11(0.94, 1.31)	0.20	
Hypertension			0.834
Yes	1.18(0.95, 1.47)	0.14	
No	1.18(0.95, 1.47)	0.13	
Diabetes			0.853
Yes	1.26(0.84, 1.88)	0.24	
No	1.21(1.02, 1.45)	0.03	

### NHHR was associated with Sarcopenia in individuals with cancer

There was a positive association between the NHHR in individuals with cancer and the prevalence of sarcopenia. This significantly positive association remained stable across all the models. In Adjusted Model 2, for every one-unit increase in the NHHR, the risk of developing sarcopenia rose by 18% (95% confidence interval [CI]: 1.01–1.39, *P* = 0.036). This relationship was further investigated by classifying the NHHR into low, medium, and high tertiles. In Adjusted Model 2, the risk of sarcopenia was 2.09 times higher in the high group compared to the low group (95% CI: 1.12–3.92, *P* = 0.022). The medium group compared to the low group did not show statistically significant differences (95% CI: 0.92–2.62, *P* = 0.101), but this difference exhibited a significant trend (*P* for trend = 0.022). High and medium NHHR levels compared to the low NHHR level were statistically significant in both the unadjusted model and Adjusted Model 1, and this difference also showed a significant trend. Analyses of cancer types with larger sample sizes showed a positive correlation between the NHHR and the occurrence of sarcopenia in individuals with breast cancer. However, a significant correlation was not found between the NHHR and the prevalence of sarcopenia in individuals with non-melanoma skin cancer, cervical and uterine cancer, or prostate cancer. In the adjusted Model 2 for individuals with breast cancer, each unit increase in the NHHR was associated with a 70% increase in the risk of sarcopenia (95% CI: 1.25–2.30, *P* = 0.001) (Table 2).

### The nonlinear relationship between NHHR and muscle index in females with cancer

**Fig. 3:**
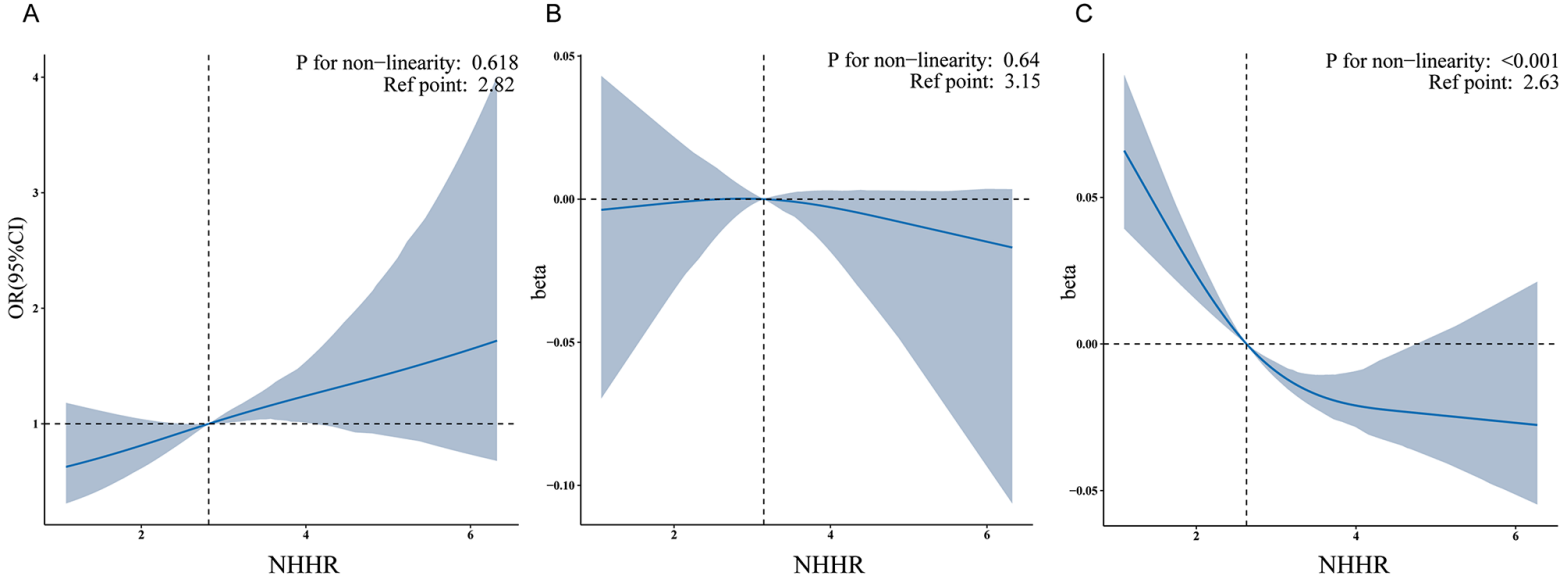
(**A**) There is a linear relationship between NHHR and the presence of sarcopenia in individuals with cancer. (**B**) There is a linear relationship between NHHR and muscle index in males with cancer. (**C**) There is a nonlinear relationship between NHHR and muscle index in females with cancer

In Adjusted Model 2, the association between the NHHR and sarcopenia, as well as muscle index, using weighted regression cubic splines was explored. The smallest and largest 2.5% of the data from the NHHR were excluded to ensure that the final data were more representative. Figure 3(A) demonstrates a positive linear association between the NHHR and sarcopenia in individuals with cancer (*P* for non-linear = 0.618). Figure 3(B) illustrates a negative linear association between the NHHR and the muscle index in males with cancer (*P* for non-linear = 0.64). Figure 3(C) shows a non-linear association between the NHHR and the muscle index in females with cancer (*P* for non-linear < 0.001). This implies that the NHHR level may have a greater effect on sarcopenia in females with cancer than in males with cancer.

### Subgroup analysis

Through subgroup analysis and interaction testing, the association between the NHHR and sarcopenia in different sub-groups was tested. Sex, age, PIR, educational attainment, moderate activity, marital status, alcohol habit, smoking habit, coronary disease, arthritis, hypertension, and diabetes were included as stratification factors in the subgroup analysis (Table 3). The results of the subgroup analysis showed that, except for sex, the other stratification factors did not show a significant interaction with the NHHR and sarcopenia (*P* for interaction > 0.05). Compared to males with cancer, a high NHHR level in females with cancer was more frequently associated with sarcopenia (OR = 1.41, 95% CI: 1.17, 1.70, *P* for interaction < 0.001). Therefore, females with cancer are more likely to be affected by changes in the NHHR level, leading to sarcopenia. This is consistent with the RCS results.

## Discussion

This study included 1,602 cancer individuals with cancer from the NHANES database and explored the potential association between the NHHR and the presence of sarcopenia. The results showed a significant positive association between an elevated NHHR in individuals with cancer and the presence of sarcopenia. There was also a significant negative association with the muscle index, especially in females. The RCS analysis further demonstrated a non-linear negative correlation. These findings were not affected by other demographic factors.

The NHHR integrates all atherogenic cholesterols and is considered to be the most useful of the numerous lipid indices. An increasing number of studies have adopted the NHHR as a sensitive indicator of the risk of lipid-related diseases [26]. This study is the first to organically combine cancer, sarcopenia, and atherogenic cholesterols, to explore the relationships between these three factors.

Epidemiological studies have indicated that individuals with different types of cancers have different rates of sarcopenia. A nationwide multicenter cohort study from China reported that the frequency of sarcopenia in individuals with ovarian, bladder, lung, prostate, and some gastrointestinal malignancies was over 25%, which is slightly higher than that in other cancers [27]. According to an analysis of the prevalence of sarcopenia in individuals with various medical conditions, sarcopenia is more common in those with gastrointestinal, lung, gynecological, and urinary system cancers [28]. The findings of this study are consistent with these epidemiological surveys, showing that among the 1,602 individuals with sarcopenia, a higher percentage had prostate, respiratory, colorectal, or ovarian cancers. This suggests that individuals with these cancers are more prone to sarcopenia, supporting the need for prognostic management and early intervention.

Previous studies identified a link between sarcopenia and atherogenic cholesterols. They have shown that decreased HDL-C and increased non-HDL-C are two important risk factors for sarcopenia [29, 30]. Individuals with sarcopenia exhibit noticeably elevated serum TC and non-HDL-C levels compared to normal individuals [19].

Many studies have indirectly confirmed that abnormalities in lipid metabolism significantly affect the prevalence of sarcopenia in individuals with cancer. Some researchers have proposed that lipid peroxidation in individuals with cancer leads to the generation of aldehydes, particularly 4-hydroxynonenal and acetaldehyde, which then form adducts with proteins involved in apoptosis, thereby accelerating molecular turnover and functional operation, ultimately leading to muscle loss [31]. Additionally, another study found that among 225 individuals with colorectal cancer, 40% had sarcopenia, and fat degeneration was more pronounced in individuals with coexisting sarcopenia than in those without sarcopenia [32]. Furthermore, a meta-analysis showed that a decrease in omega-3 polyunsaturated fatty acids leads to sarcopenia [33].

The mechanism underlying the association between sarcopenia and lipid metabolism in individuals with cancer remains unknown. Some researchers have evaluated the biological differences in muscle atrophy and fat infiltration in pancreatic cancer and periampullary adenocarcinoma and found that diabetes-like changes in lipid metabolism were particularly pronounced in individuals with low muscle density. They suggested that cancer-related muscle atrophy may be correlated with lipid metabolism damage, potentially due to insulin insensitivity [34]. On the other hand, inflammation may be a potential mechanism linking disorders of lipid metabolism with the coexistence of cancer and sarcopenia. Studies have found that genetic deletion or drug-induced inhibition of interleukin 6 or its receptor can partially protect adipose tissue and skeletal muscle from atrophy. Conversely, a high circulating level of interleukin 6 caused by a cytokine storm can lead to abnormal lipid metabolism and muscle loss in mice with soft tissue sarcoma [35]. Furthermore, higher plasma pro-inflammatory markers sTNF-R1 and CHI3L1, lipid metabolic abnormalities, sarcopenia, and risk of tumor recurrence were found to be concurrently associated based on multi-omics studies examining the main pathways connecting sarcopenia and liver cancer. Among them, the CHI3L1 level was negatively correlated with TC and considerably impairs cholesterol metabolism in individuals with cancer and coexisting sarcopenia [36]. Additionally, the activation of the ferroptosis pathway in cancer is a common mechanism leading to cholesterol metabolism and sarcopenia [37, 38], indicating that ferroptosis may also be a potential pathway linking lipid metabolism to sarcopenia.

This study found that sex is an important stratifying factor, with females having a more significant association. This is consistent with previous *studies* indicating that sarcopenia is more likely to occur in females with cancer [39]. The potential mechanism by which sarcopenia in females with cancer is more sensitive to changes in the NHHR may be related to estrogen. Muscle satellite cells proliferate and differentiate in response to estrogen, thereby increasing muscle mass and strength [40]. Individuals with cancer often exhibit decreased hormone levels [41]. When estrogen is deficient, the IGF1 signaling pathway is suppressed, while the Akt/FoxO and Akt/mTOR pathways are activated, leading to the disruption of muscle protein homeostasis. As a result, there is a change from protein synthesis to protein breakdown, which causes muscle fiber atrophy and leads to a gradual decrease in muscle mass and strength [42]. In addition, sex chromosome genes in females with cancer are more prone to losing their stability compared to males. This increases the likelihood of a mitochondrial metabolic imbalance and impaired muscle oxidation capacity in females, ultimately making them more susceptible to muscle atrophy [43, 44]. This study further analyzed several types of cancer with relatively larger sample sizes among the 1,602 individuals with cancer and sarcopenia, and discovered an important connection between sarcopenia and the NHHR in individuals with breast cancer. Previous studies have found abnormal levels of TC and HDL-C in the serum of individuals with breast cancer [45]; however, it is still debatable whether a high HDL-C level can prevent breast cancer [46]. Some studies have found that proliferating breast cancer cells increase nutritional stress within the tumor microenvironment, leading to overexpression of fat metabolism-related genes and increased fatty acid synthesis, ultimately resulting in abnormal lipid metabolism [47]. Chemotherapy and endocrine therapy for breast cancer can disrupt lipid metabolism to varying degrees [48, 49] and one of the main outcomes of lipid metabolism disorders is the promotion of sarcopenia [50].

### Strengths and limitations

Compared to previous studies, this study utilized a weighted analysis of data from the NHANES database with a larger and more representative sample size, providing an objective reflection of the real situation among the US population. Controlling for potential confounding covariates and conducting subgroup analyses on the stratified variables further enhanced the integrity and reliability of the conclusions. This study explored the relationship between the NHHR and sarcopenia combined with cancer, which is innovative and holds a significant reference value for clinical monitoring and prevention. Additionally, the study identified a more significant non-linear U-shaped association between the muscle index and the NHHR among females with cancer, providing clearer guidance for the clinical prevention of sarcopenia.

This study had several limitations. First, a causal relationship cannot be established using a cross-sectional design, which only reveals potential correlations. Additionally, this study was conducted on individuals with cancer without considering the significant differences between different cancer cohorts. Furthermore, this study could not compare the time of cancer diagnosis and sarcopenia diagnosis. Moreover, it could not be confirmed whether sarcopenia is a manifestation of cancer-related cachexia. These factors limited the precision and depth of this study. As there are insufficient pertinent clinical staging data, this study was limited in its ability to investigate the association from the perspective of the clinical tumor burden. Therefore, further validation is required through prospective studies with larger sample sizes, targeting groups with specific cancers, and including more detailed clinical information.

## Conclusion

The results showed a positive association between the NHHR and the presence of sarcopenia among individuals with cancer as well as identifying a non-linear association between the NHHR and the muscle index in females with cancer, suggesting that the NHHR may serve as an effective marker for sarcopenia risk among individuals with cancer. Enhancing lipid metabolism and maintaining the NHHR level within a specific range could help lower the risk of sarcopenia among individuals with cancer, particularly females, and mitigate the negative impacts of sarcopenia.

## Data Availability

This study utilized publicly available NHANES data on the American population, which can be accessed through the following website: (https://wwwn.cdc.gov/nchs/nhanes/analyticguidelines.aspx).

## Abbreviations

BMI: Body mass index
CI: Confidence Interval
DXA: Dual-energy X-ray absorptiometry
HDL-C: High-density lipoprotein cholesterol
LDL-C: Low-density lipoprotein cholesterol
NHANES: National Health and Nutrition Examination Survey
NHHR: Non-HDL-C and HDL-C ratio
PIR: Poverty-to-income ratio
RCS: Regression cubic splines
TC: Total Cholesterol
US: United States
